# Author Correction: A virtual rodent predicts the structure of neural activity across behaviours

**DOI:** 10.1038/s41586-025-09407-y

**Published:** 2025-08-11

**Authors:** Diego Aldarondo, Josh Merel, Jesse D. Marshall, Leonard Hasenclever, Ugne Klibaite, Amanda Gellis, Yuval Tassa, Greg Wayne, Matthew Botvinick, Bence P. Ölveczky

**Affiliations:** 1 Department of Organismic and Evolutionary Biology and Center for Brain Science, Harvard University, Cambridge, MA USA; 2 DeepMind, Google, London, UK; 3 Gatsby Computational Neuroscience Unit, University College London, London, UK; 4 Present Address: Fauna Robotics, New York, NY USA; 5 Present Address: Reality Labs, Meta, New York, NY USA

**Keywords:** Motor control, Network models, Biophysical models

Correction to: *Nature* 10.1038/s41586-024-07633-4 Published online 11 June 2024

Following publication of this article, we discovered a bug in the code that extracted and sorted spikes from the neural recordings. While the sorting itself was good, the final step in the analysis pipeline inadvertently introduced short periodic dropout of spikes for a fraction of the cells, causing the cell quality filters we used to exclude units that were otherwise well-tracked. We have fixed the code and reanalyzed the recordings, which now include *N* = 2,654 and *N* = 1,177 putative single units for the dorsal lateral striatum and motor cortex, respectively (previously, these numbers were 1,249 and 843; edits to values made in the sixth paragraph of main text). In the Fig. 3c legend, the number of neurons predicted now lists 1,788 neurons in DLS and 1,095 neurons in MC, versus 732 and 769, respectively, in the original. None of this had any impact on the findings or conclusions of our paper. Indeed, all our results remain unchanged, with effect sizes comparable to those in the original publication. Figures 3, 4 and Extended Data Figs. 1, 4–10 have been updated. For explanation of changes and comparison to original figures, see the Supplementary Information accompanying this amendment.

**Supplementary information** is available in the online version of this amendment.

## Supplementary information

Notes on edits; original Figs. 3, 4 and Extended Data Figs. 1, 4–10.

