# Supplementary material for: Author Correction: A virtual rodent predicts the structure of neural activity across behaviours
Source: Nature. 2025 Aug 11;645(8079):E1. doi: 10.1038/s41586-025-09407-y (PMC12408354; doi:10.1038/s41586-025-09407-y)
Supplement: Supplementary file 1 — Notes on edits; original Figs. 3, 4 and Extended Data Figs. 1, 4–10. [file 41586_2025_9407_MOESM1_ESM.pdf]

nature portfolio

https://doi.org/10.1038/s41586-025-09407-y

Supplementary information

Author Correction: A virtual rodent predicts the structure of neural activity across behaviours

In the format provided by the authors and unedited

Main article

Text

One sentence in the main text denoting the total number of neurons used throughout the analyses has been updated as a part of this correction.

New text is on the top; old text is on the bottom

To this end, we recorded the behavior of freely-moving rats in a circular arena with an array of six cameras while measuring neural activity from the DLS or MC (DLS: 3 animals, 353.5 hours, 2654 neurons; MC: 3 animals, 253.5 hours, 1177 neurons) with custom 128-channel tetrode drives (Fig. 1 C, Extended Data Fig. 1).

To this end, we recorded the behavior of freely-moving rats in a circular arena with an array of six cameras while measuring neural activity from the DLS or MC (DLS: 3 animals, 353.5 hours, 1249 neurons; MC: 3 animals, 253.5 hours, 843 neurons) with custom 128-channel tetrode drives (Fig. 1 C, Extended Data Fig. 1).

Figure 3

The spike raster plot in panel A has been updated to include the corrected data. As in the original, the neurons are sorted via hierarchical clustering on smoothed firing rates, thus resulting in a distinct sorting. Panels B, C, and D have been updated to include the corrected data, with no notable changes to the results.

Caption

New text is on the top; old text is on the bottom

Data include neurons significantly predicted by each GLM (Benjamini–Hochberg corrected Wilcoxon signedrank test, $\alpha = 0.05$) from a total of N = 1788 neurons in DLS and 1095 neurons in MC.

Data include neurons significantly predicted by each GLM (Benjamini–Hochberg corrected Wilcoxon signedrank test, $\alpha = 0.05$) from a total of N = 732 neurons in DLS and 769 neurons in MC.

The original figure appears on top, while the corrected figure appears on the bottom.

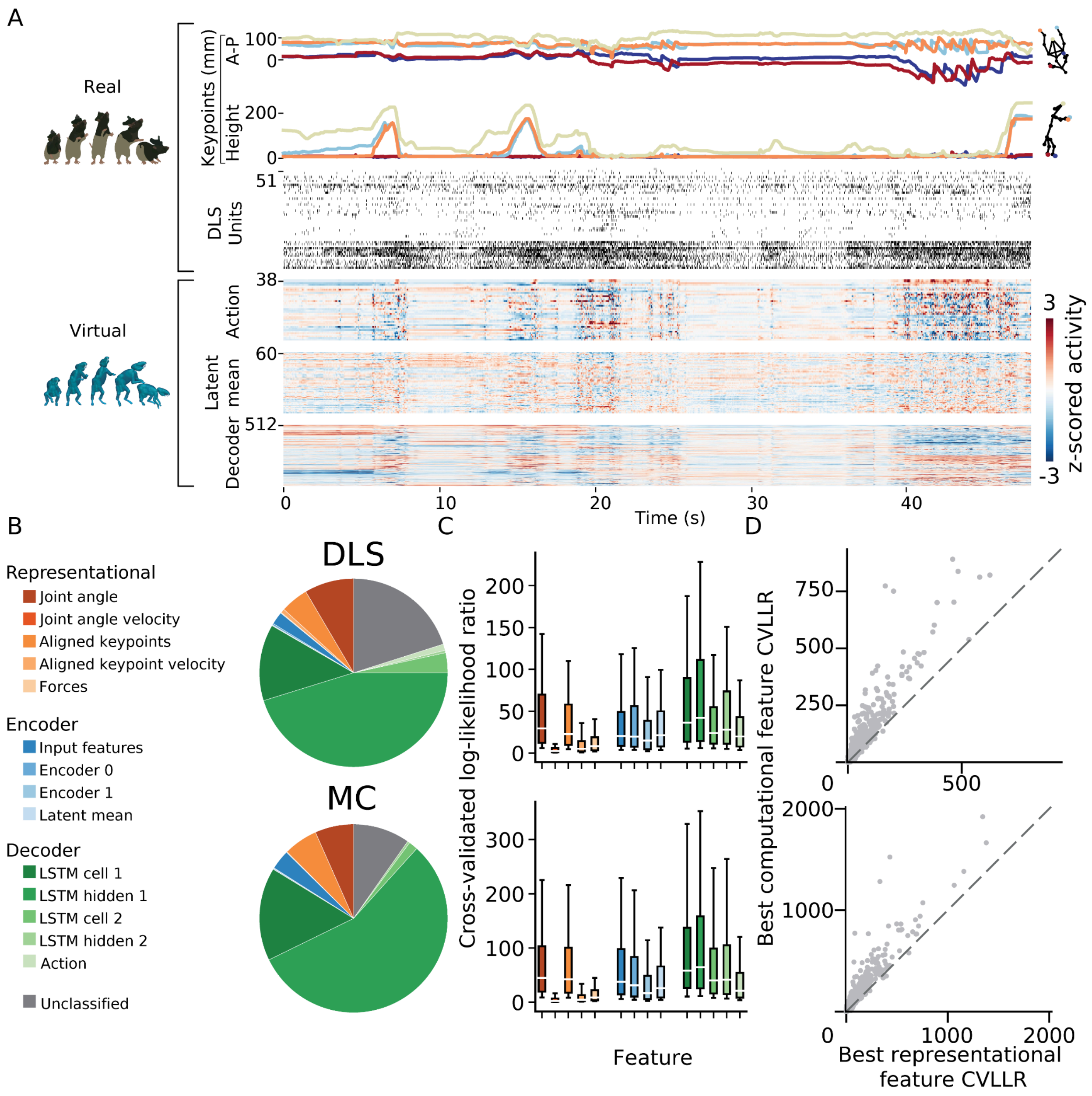
A
Real
Virtual
Keypoints (mm)
A-P
Height
DLS Units
Action
Latent mean
Decoder
z-scored activity
Time (s)
B
Representational
Joint angle
Joint angle velocity
Aligned keypoints
Aligned keypoint velocity
Forces
Encoder
Input features
Encoder 0
Encoder 1
Latent mean
Decoder
LSTM cell 1
LSTM hidden 1
LSTM cell 2
LSTM hidden 2
Action
Unclassified
DLS
MC
C
Cross-validated log-likelihood ratio
Feature
D
Best computational feature CVLLR
Best representational feature CVLLR

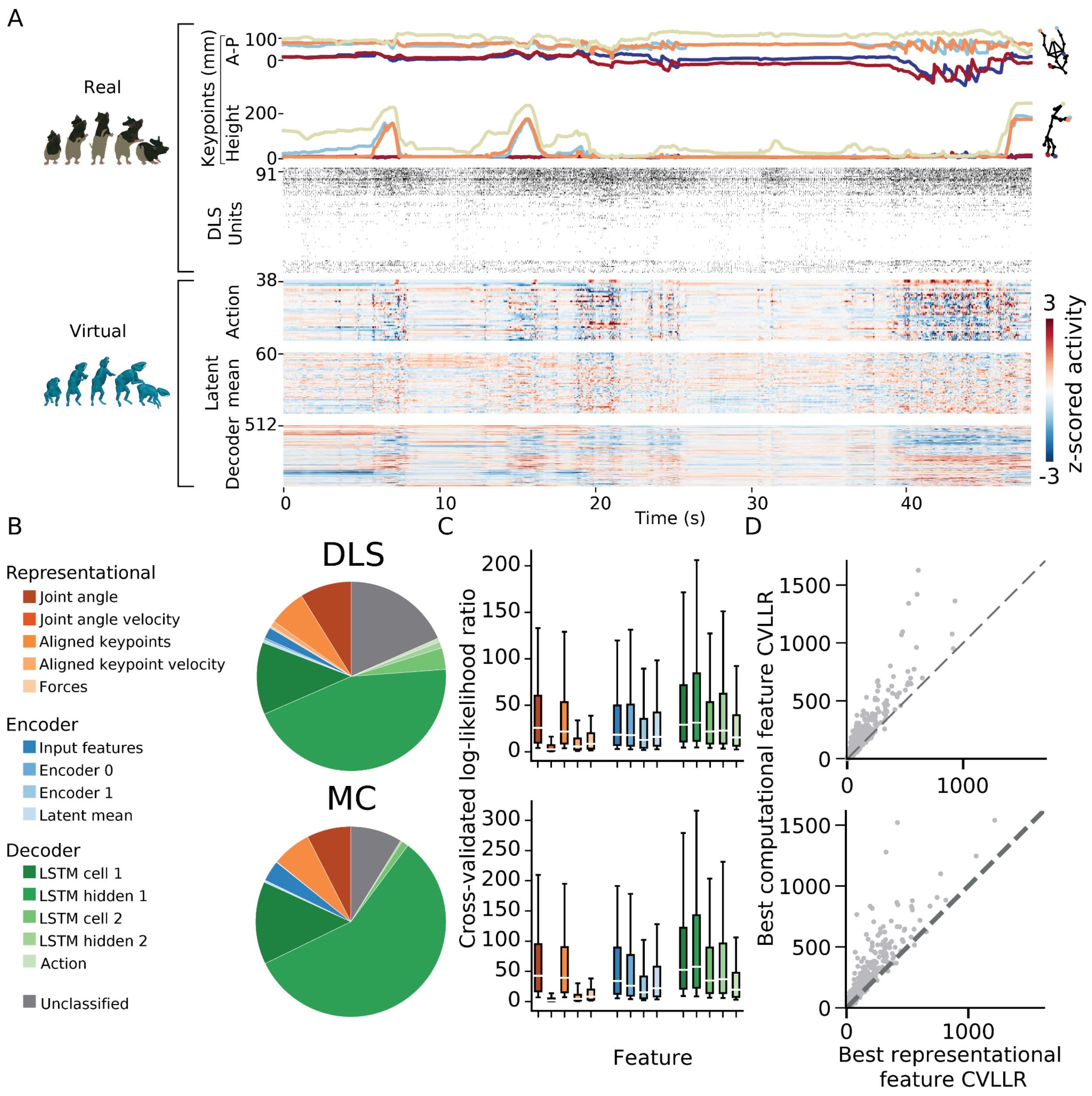
A
Real
Virtual
Keypoints (mm)
A-P
Height
100
0
200
DLS Units
91
Action
38
Latent mean
60
Decoder
512
z-scored activity
3
-3
0
10
20
30
40
Time (s)
B
Representational
Joint angle
Joint angle velocity
Aligned keypoints
Aligned keypoint velocity
Forces
Encoder
Input features
Encoder 0
Encoder 1
Latent mean
Decoder
LSTM cell 1
LSTM hidden 1
LSTM cell 2
LSTM hidden 2
Action
Unclassified
DLS
MC
C
Cross-validated log-likelihood ratio
Feature
D
Best computational feature CVLLR
Best representational feature CVLLR

Figure 4

All panels have been updated to include the corrected data. As in the originals, we used hierarchical clustering in panels A and B to respectively sort neurons as a function of their average firing rates across behavior, and sort behaviors in representational dissimilarity matrices. This resulted in a visually distinct sorting from the original. There were no notable changes to the results.

The original figure appears on top, while the corrected figure appears on the bottom.

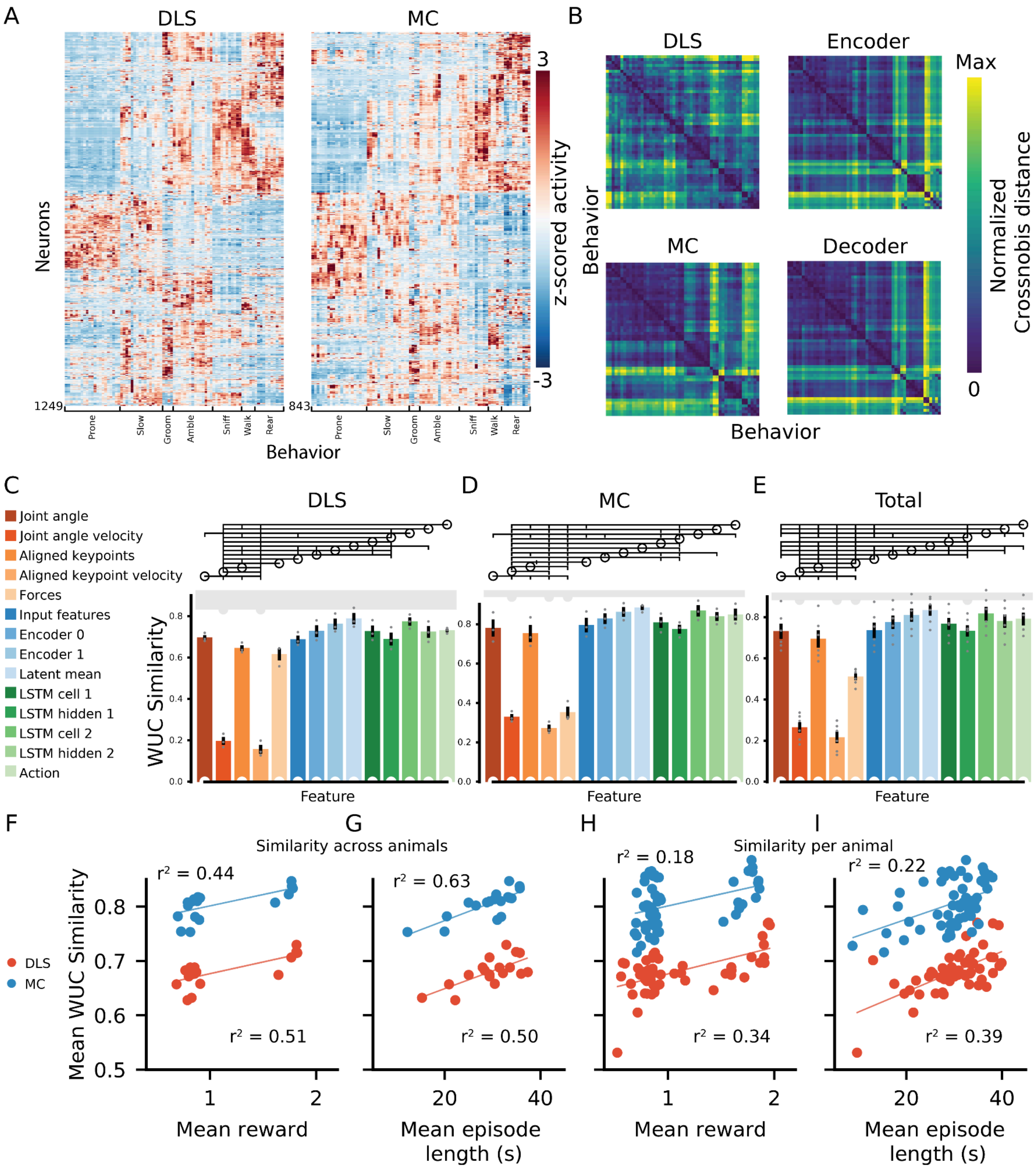

A
DLS
MC
Neurons
1249
843
Prone
Slow
Groom
Amble
Sniff
Walk
Rear
Behavior
3
-3
z-scored activity
B
DLS
Encoder
MC
Decoder
Behavior
Max
0
Normalized
Crossnobis distance
C
D
E
Total
Joint angle
Joint angle velocity
Aligned keypoints
Aligned keypoint velocity
Forces
Input features
Encoder 0
Encoder 1
Latent mean
LSTM cell 1
LSTM hidden 1
LSTM cell 2
LSTM hidden 2
Action
WUC Similarity
Feature
F
G
H
I
Similarity across animals
Similarity per animal
Mean WUC Similarity
DLS
MC
r² = 0.44
r² = 0.51
r² = 0.63
r² = 0.50
r² = 0.18
r² = 0.34
r² = 0.22
r² = 0.39
Mean reward
Mean episode length (s)

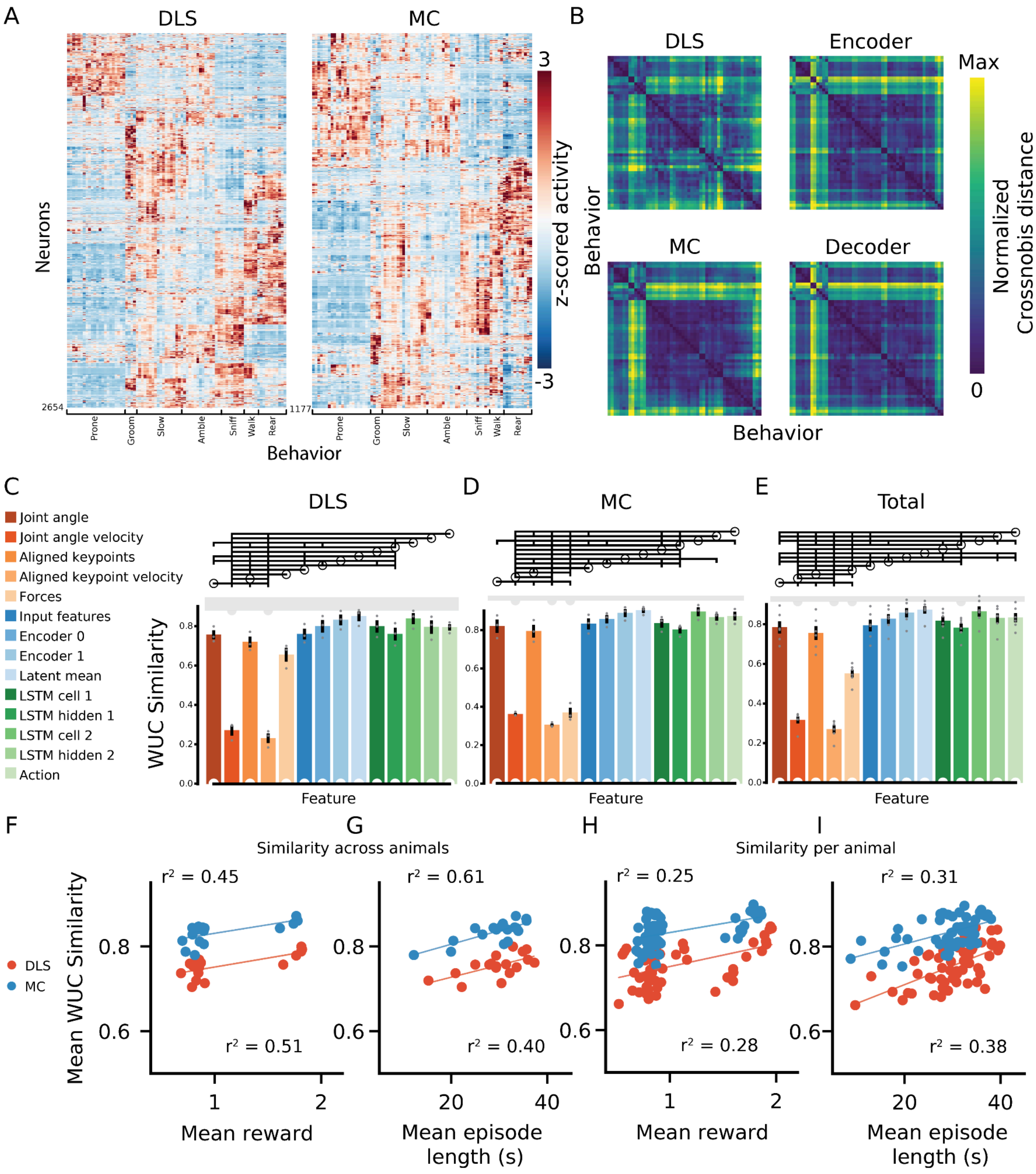

A
DLS
MC
Neurons
z-scored activity
3
-3
2654
1177
Prone
Groom
Slow
Amble
Sniff
Walk
Rear
Behavior
B
DLS
Encoder
MC
Decoder
Max
0
Normalized Crossnobis distance
Behavior
C
D
E
DLS
MC
Total
Joint angle
Joint angle velocity
Aligned keypoints
Aligned keypoint velocity
Forces
Input features
Encoder 0
Encoder 1
Latent mean
LSTM cell 1
LSTM hidden 1
LSTM cell 2
LSTM hidden 2
Action
WUC Similarity
Feature
F
G
H
I
Similarity across animals
Similarity per animal
DLS
MC
Mean WUC Similarity
r² = 0.45
r² = 0.51
r² = 0.61
r² = 0.40
r² = 0.25
r² = 0.28
r² = 0.31
r² = 0.38
Mean reward
Mean episode length (s)

Extended Data

Extended Data Figure 1

Panel D has been updated to include the corrected data.

The original figure appears on top, while the corrected figure appears on the bottom.

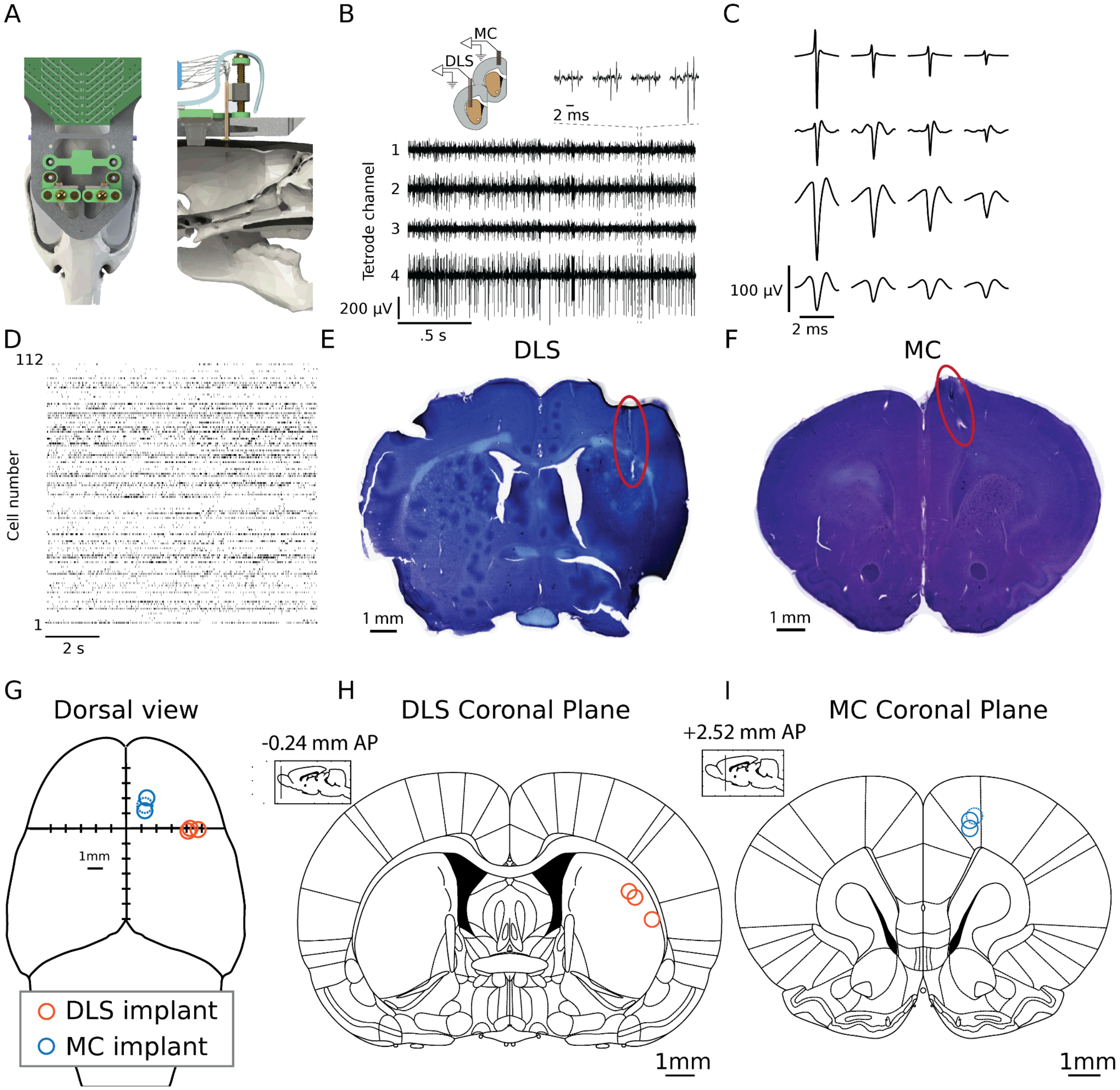
A
B
MC
DLS
2 ms
1
2
3
4
Tetrode channel
200 µV
.5 s
C
100 µV
2 ms
D
112
Cell number
1
2 s
E
DLS
1 mm
F
MC
1 mm
G
Dorsal view
1mm
DLS implant
MC implant
H
DLS Coronal Plane
-0.24 mm AP
1mm
I
MC Coronal Plane
+2.52 mm AP
1mm

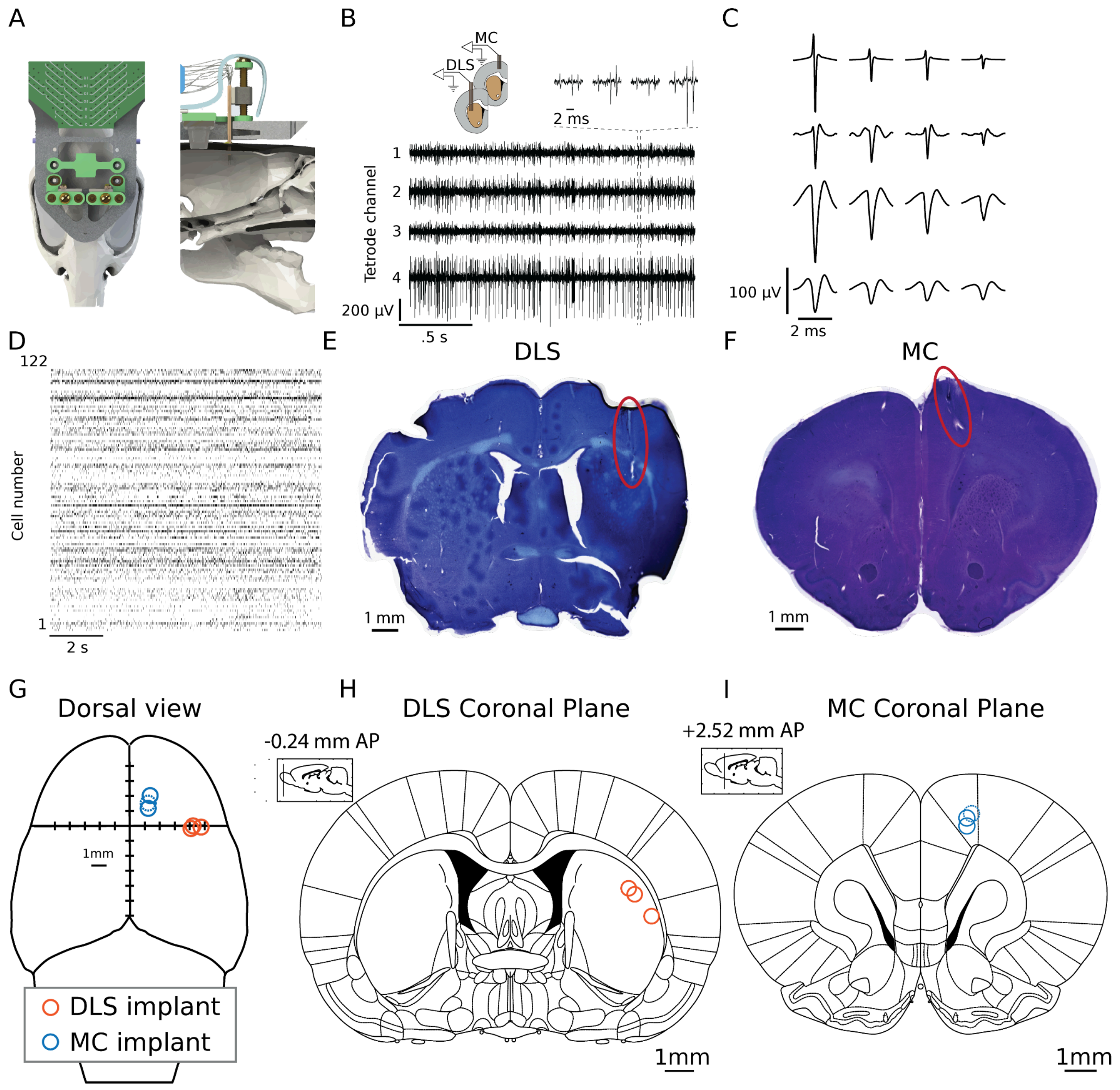

A
B
MC
DLS
2 ms
Tetrode channel
1
2
3
4
200 µV
.5 s
C
100 µV
2 ms
D
122
Cell number
1
2 s
E
DLS
1 mm
F
MC
1 mm
G
Dorsal view
1mm
DLS implant
MC implant
H
DLS Coronal Plane
-0.24 mm AP
1mm
I
MC Coronal Plane
+2.52 mm AP
1mm

Extended Data Figure 4

All panels have been updated to include the corrected data. The proportion of neurons preferentially encoding the Cartesian position and joint angles related to the head has increased relative to the original figure. The distribution of pseudo r-squared values across neurons in our encoding analyses remains similar to the original figure.

The original figure appears on top, while the corrected figure appears on the bottom.

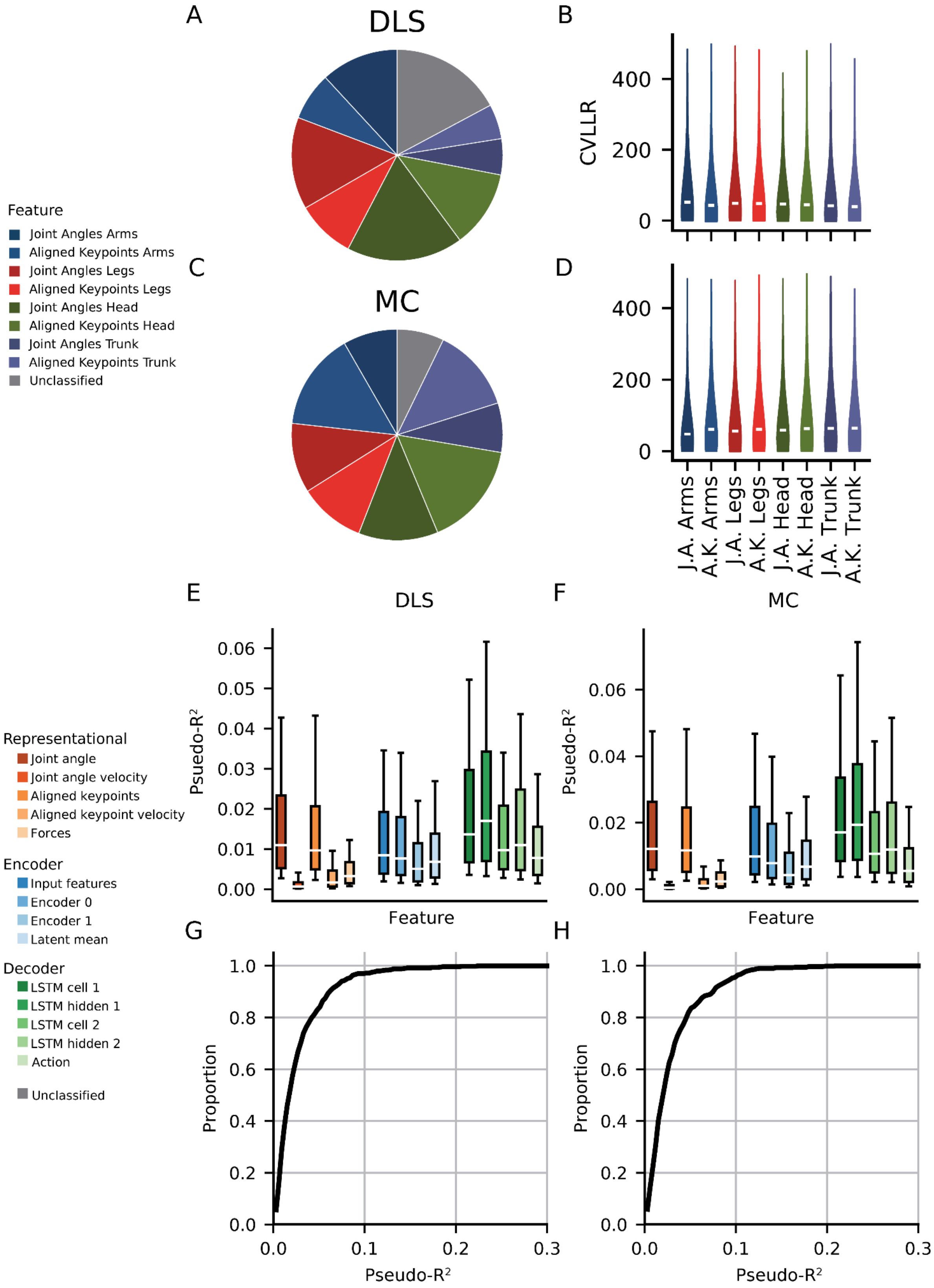

A
DLS
B
CVLLR
400
200
0
C
MC
D
400
200
0
J.A. Arms
A.K. Arms
J.A. Legs
A.K. Legs
J.A. Head
A.K. Head
J.A. Trunk
A.K. Trunk
Feature
Joint Angles Arms
Aligned Keypoints Arms
Joint Angles Legs
Aligned Keypoints Legs
Joint Angles Head
Aligned Keypoints Head
Joint Angles Trunk
Aligned Keypoints Trunk
Unclassified
E
DLS
F
MC
Psuedo-R²
Feature
Representational
Joint angle
Joint angle velocity
Aligned keypoints
Aligned keypoint velocity
Forces
Encoder
Input features
Encoder 0
Encoder 1
Latent mean
Decoder
LSTM cell 1
LSTM hidden 1
LSTM cell 2
LSTM hidden 2
Action
Unclassified
G
H
Proportion
Pseudo-R²
0.0
0.1
0.2
0.3

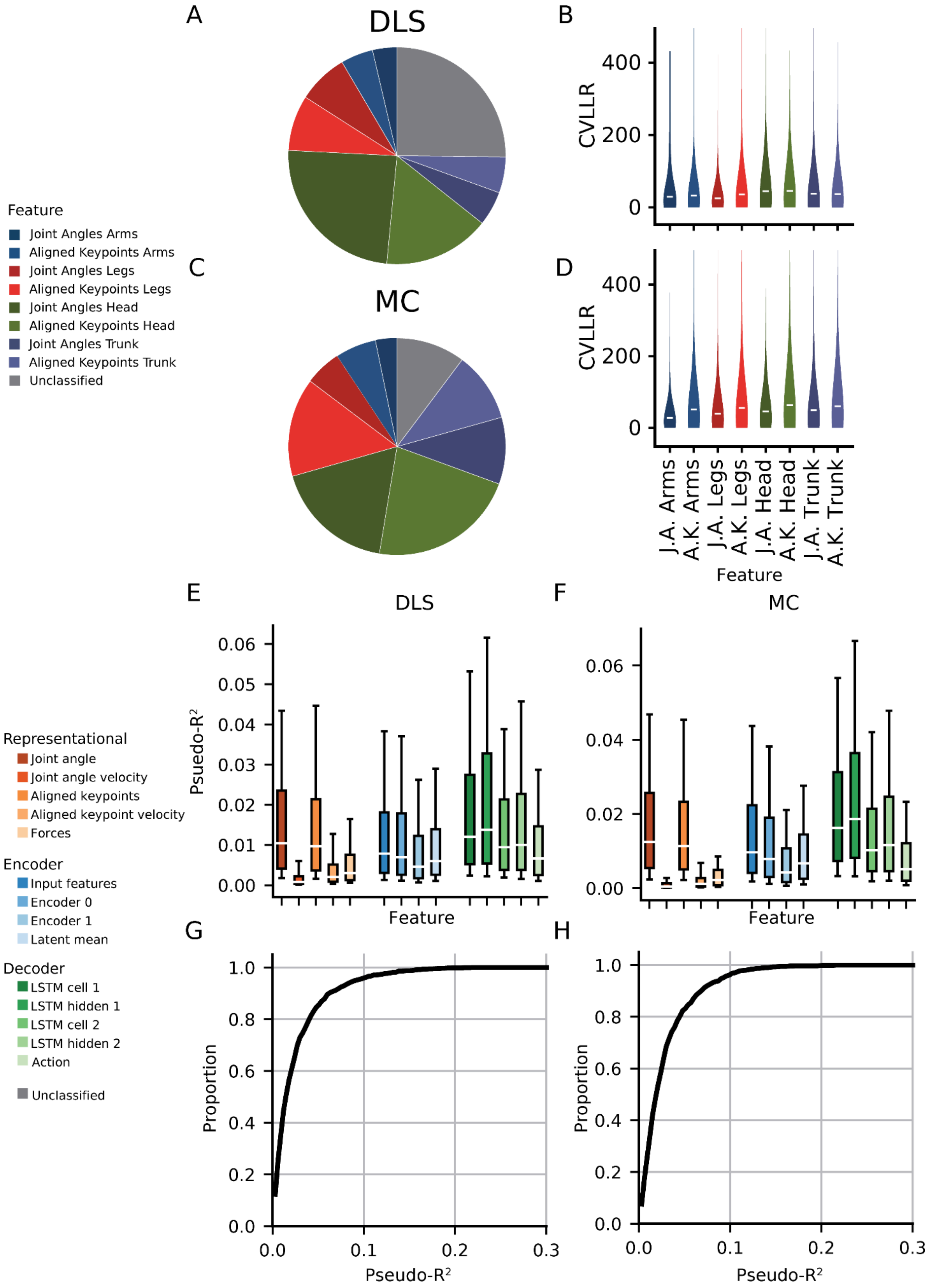

A
DLS
B
CVLLR
400
200
0
C
MC
D
CVLLR
J.A. Arms
A.K. Arms
J.A. Legs
A.K. Legs
J.A. Head
A.K. Head
J.A. Trunk
A.K. Trunk
Feature
Feature
Joint Angles Arms
Aligned Keypoints Arms
Joint Angles Legs
Aligned Keypoints Legs
Joint Angles Head
Aligned Keypoints Head
Joint Angles Trunk
Aligned Keypoints Trunk
Unclassified
E
DLS
F
MC
Pseudo-R²
Feature
Representational
Joint angle
Joint angle velocity
Aligned keypoints
Aligned keypoint velocity
Forces
Encoder
Input features
Encoder 0
Encoder 1
Latent mean
Decoder
LSTM cell 1
LSTM hidden 1
LSTM cell 2
LSTM hidden 2
Action
Unclassified
G
H
Proportion
Pseudo-R²

Extended Data Figure 5

All panels have been updated to include the corrected data. In panels A, B, and C, the proportions of neurons best predicted by different representational and computational features remains similar to the original figure. In panels D-I the distribution of cross-validated log-likelihood ratios for each feature remains similar to the original figure, as do the relative predictivity of different features.

The original figure appears on top, while the corrected figure appears on the bottom.

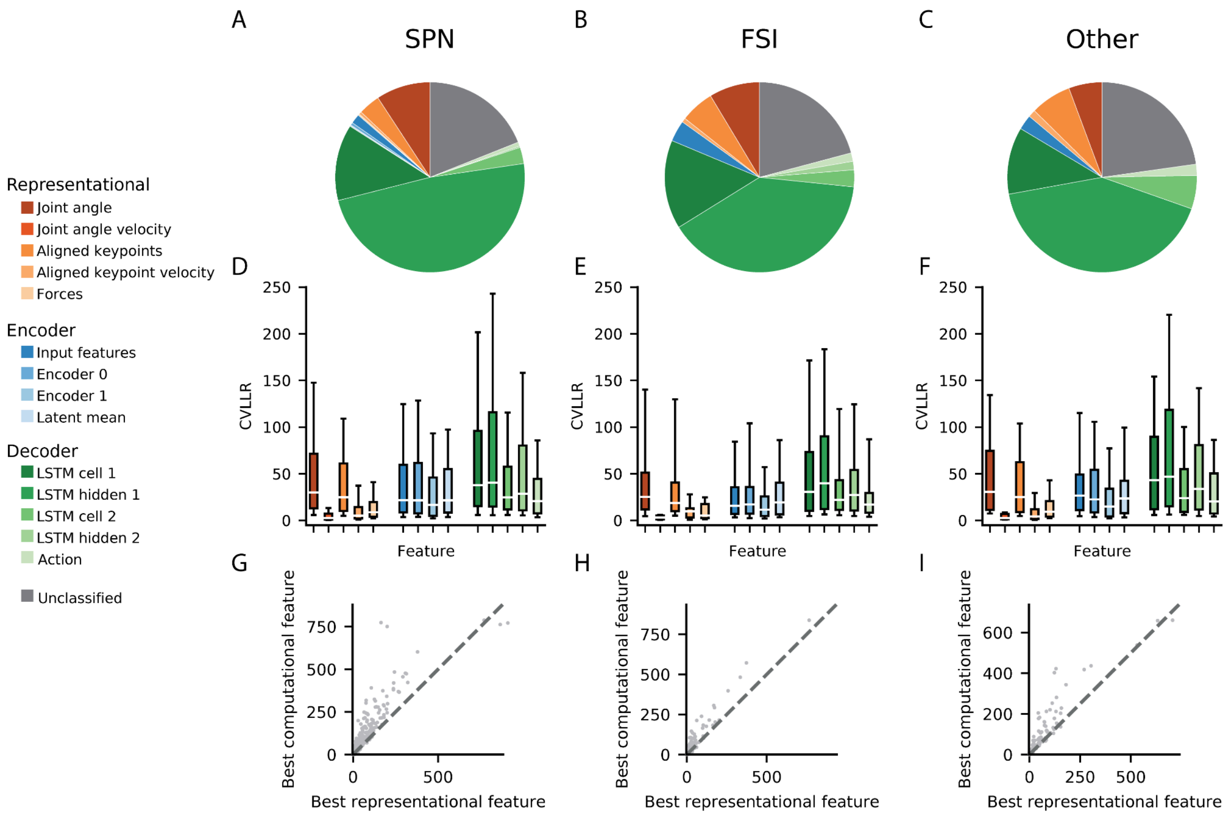
A
SPN
B
FSI
C
Other
Representational
Joint angle
Joint angle velocity
Aligned keypoints
Aligned keypoint velocity
Forces
Encoder
Input features
Encoder 0
Encoder 1
Latent mean
Decoder
LSTM cell 1
LSTM hidden 1
LSTM cell 2
LSTM hidden 2
Action
Unclassified
D
E
F
CVLLR
Feature
G
H
I
Best computational feature
Best representational feature

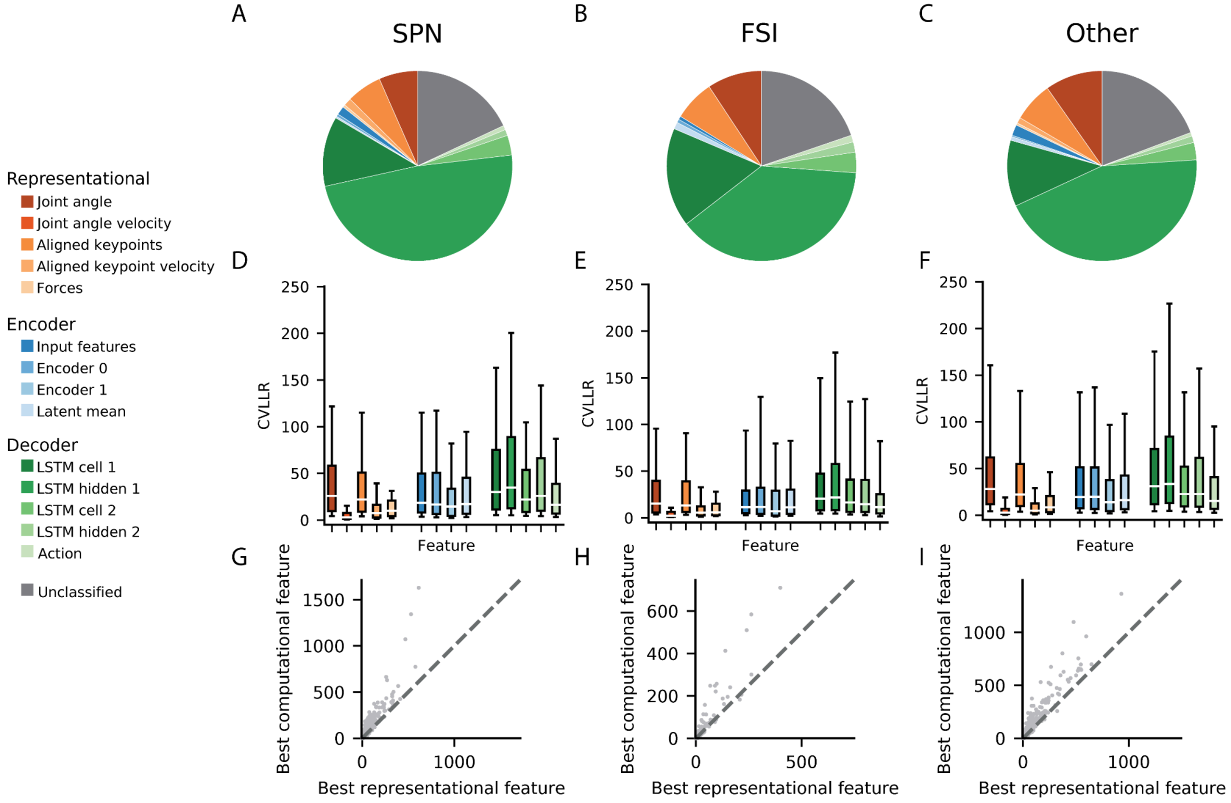
A
SPN
B
FSI
C
Other
Representational
Joint angle
Joint angle velocity
Aligned keypoints
Aligned keypoint velocity
Forces
Encoder
Input features
Encoder 0
Encoder 1
Latent mean
Decoder
LSTM cell 1
LSTM hidden 1
LSTM cell 2
LSTM hidden 2
Action
Unclassified
D
E
F
CVLLR
Feature
G
H
I
Best computational feature
Best representational feature

Extended Data Figure 6

All panels have been updated to include the corrected data. In panels A, C, E, and G, the distribution of peak encoding as a function of temporal offset remains similar to the original figure for all cell types. In panel B, the peak average cross-validated log-likelihood ratio (CVLLR) changed from -200 ms to -100 ms for spiny projection neurons predicted using joint angles. In panel D, the peak average CVLLR changed from -100ms to 0ms for unclassified neurons predicted using the inverse dynamics model features.

The original figure appears on top, while the corrected figure appears on the bottom.

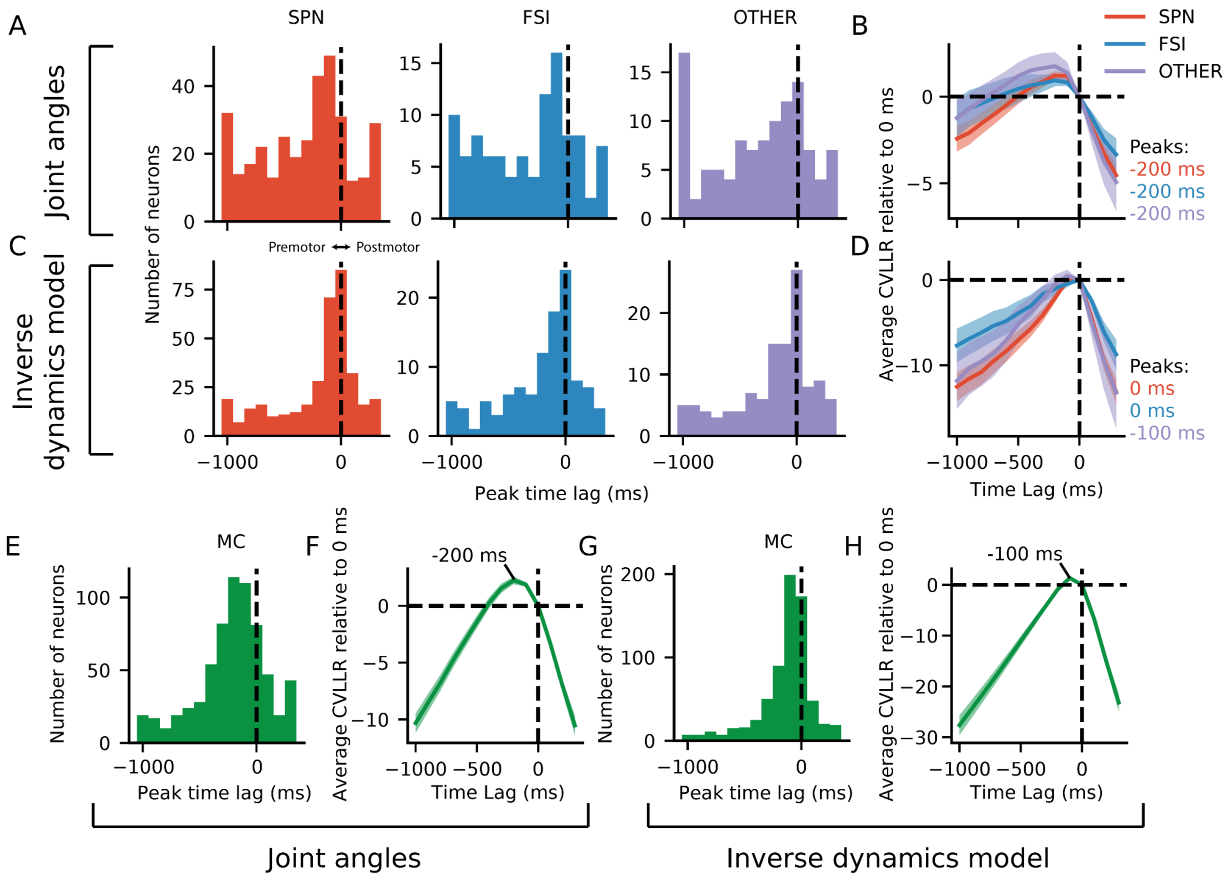

A
SPN
FSI
OTHER
B
Joint angles
Number of neurons
Premotor ↔ Postmotor
C
Inverse dynamics model
D
Peak time lag (ms)
Average CVLLR relative to 0 ms
Time Lag (ms)
Peaks:
-200 ms
-200 ms
-200 ms
Peaks:
0 ms
0 ms
-100 ms
E
MC
F
-200 ms
G
MC
H
-100 ms
Joint angles
Inverse dynamics model

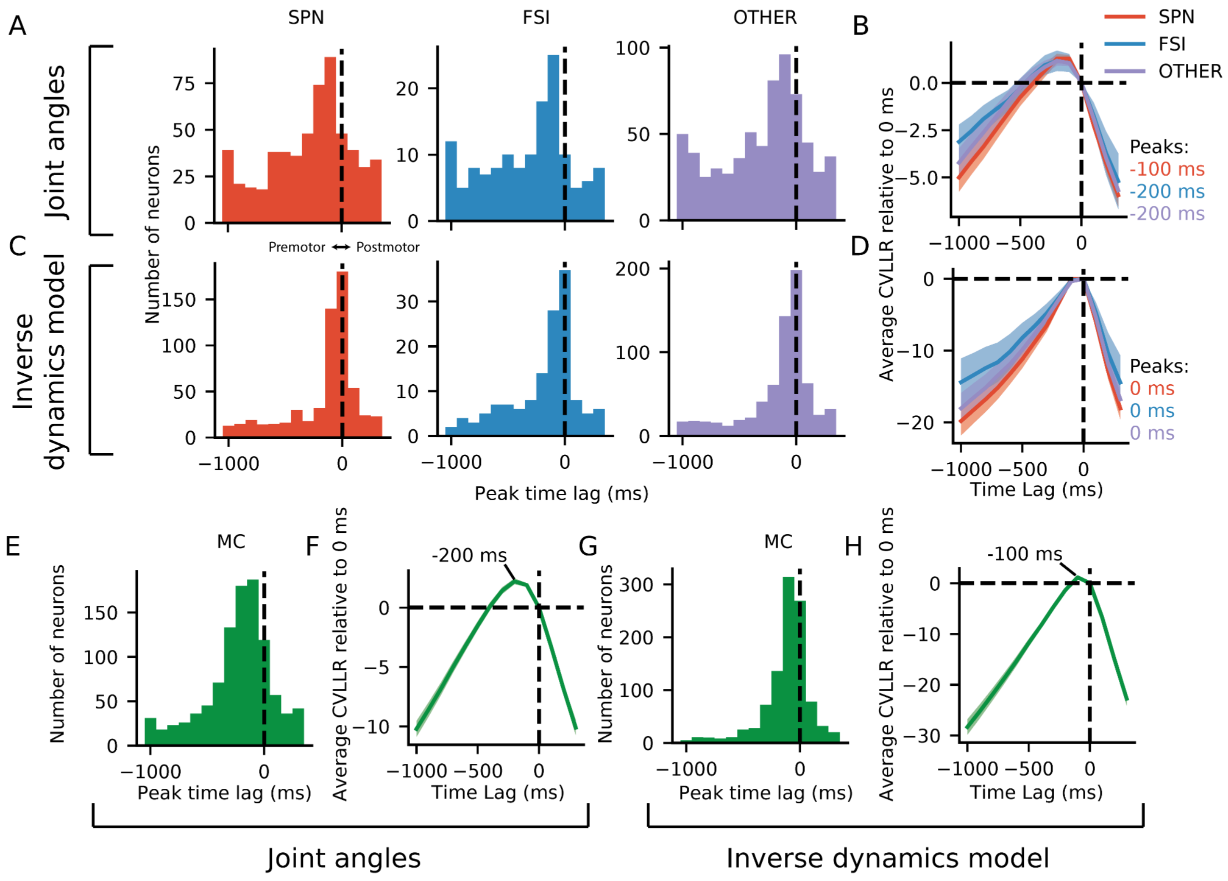
A
B
C
D
E
F
G
H
SPN
FSI
OTHER
Joint angles
Inverse dynamics model
Number of neurons
Premotor
Postmotor
Peak time lag (ms)
Average CVLLR relative to 0 ms
Time Lag (ms)
Peaks:
-100 ms
-200 ms
-200 ms
0 ms
0 ms
0 ms
MC
-200 ms
-100 ms

Extended Data Figure 7

Panels C, D, E, and F have been updated to include the corrected data. In panel C, the proportion of neurons that were better predicted by the inverse dynamics model trained on a body of the correct mass than on trained on a body of 1.1 times the correct mass is now significantly different. In panel F, one of the multiple comparisons tests between the representational similarity with motor cortical activity of inverse dynamics models trained to control bodies of different masses is no longer significantly different.

The original figure appears on top, while the corrected figure appears on the bottom.

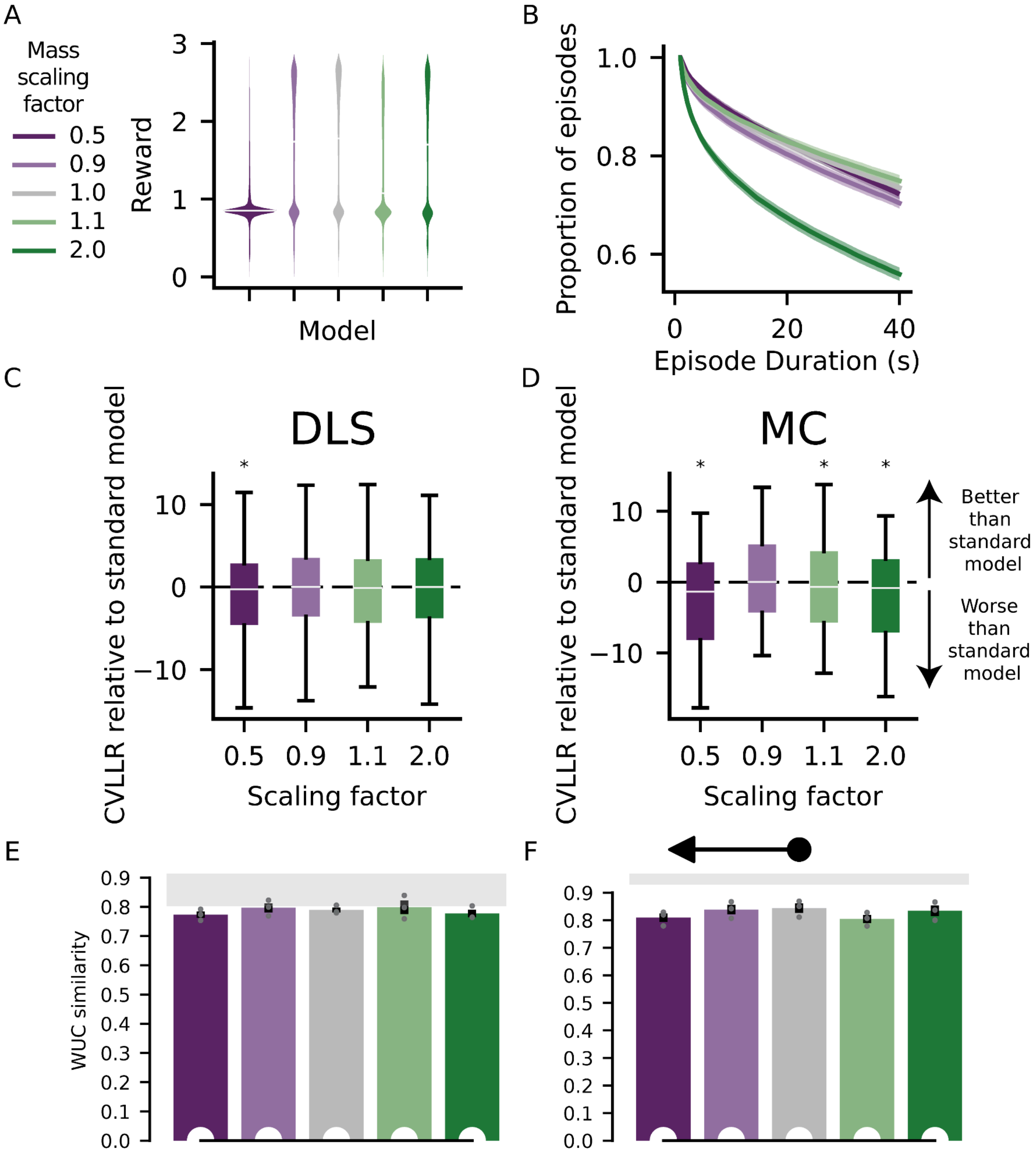
A
Mass scaling factor
0.5
0.9
1.0
1.1
2.0
Reward
Model
B
Proportion of episodes
Episode Duration (s)
C
DLS
CVLLR relative to standard model
Scaling factor
D
MC
Better than standard model
Worse than standard model
E
WUC similarity
F

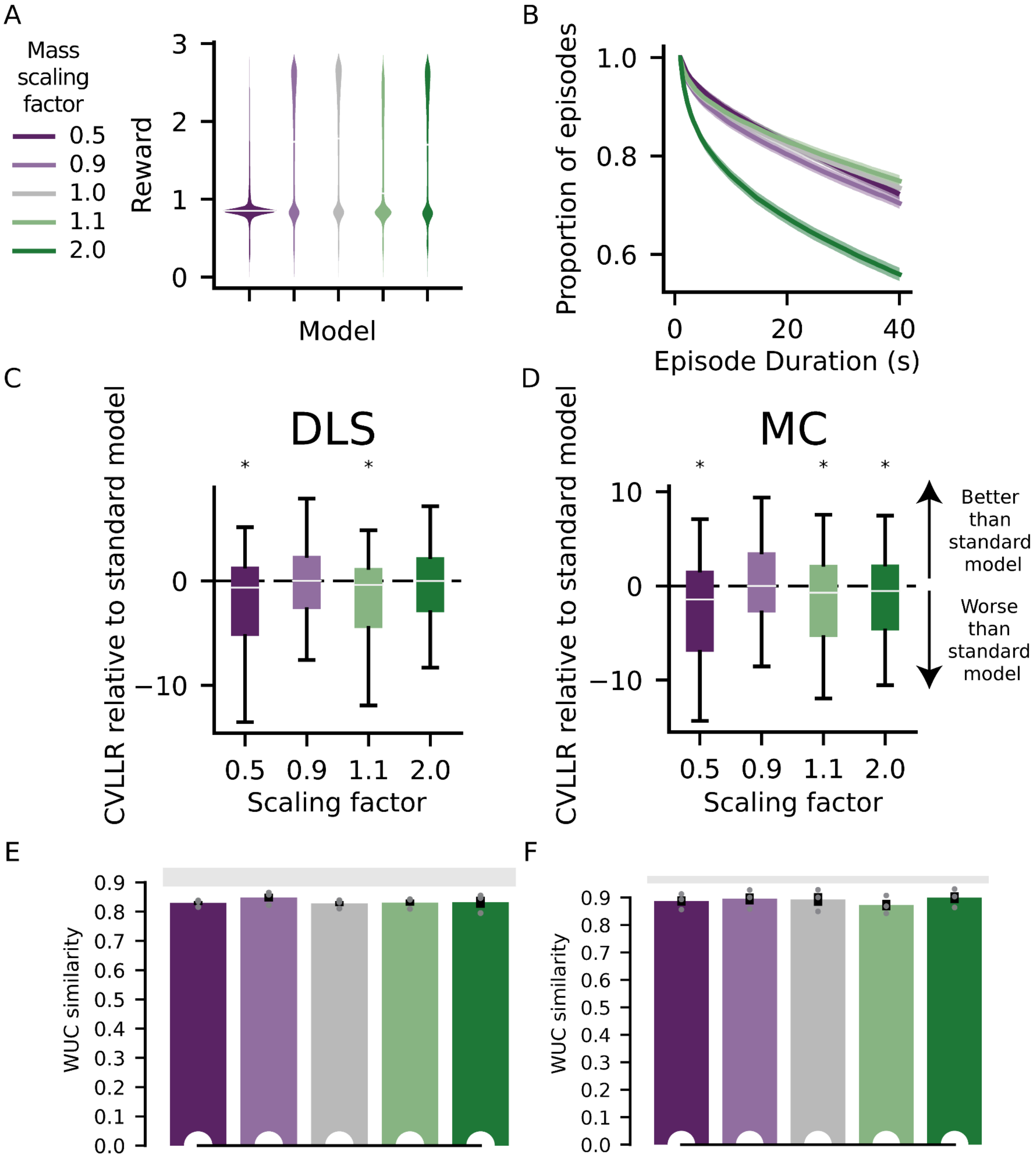
A
Mass scaling factor
0.5
0.9
1.0
1.1
2.0
Reward
Model
B
Proportion of episodes
Episode Duration (s)
C
DLS
CVLLR relative to standard model
Scaling factor
D
MC
Better than standard model
Worse than standard model
E
WUC similarity
F

Extended Data Figure 8

Panels C, D, E, and F have been updated to include the corrected data. In panels C and D, there are minor differences in the proportion of neurons that were better predicted by the inverse dynamics model trained on a body of the correct relative mass of the head to one trained on bodies with different relative head masses. Namely, the comparison between models trained with 90% of the correct relative head mass and those trained with the correct relative head mass is no longer significant in the striatum. Similarly, the comparison between models trained with 110% of the correct relative head mass and those trained with the correct relative head mass is now significant in both the striatum and motor cortex.

The original figure appears on top, while the corrected figure appears on the bottom.

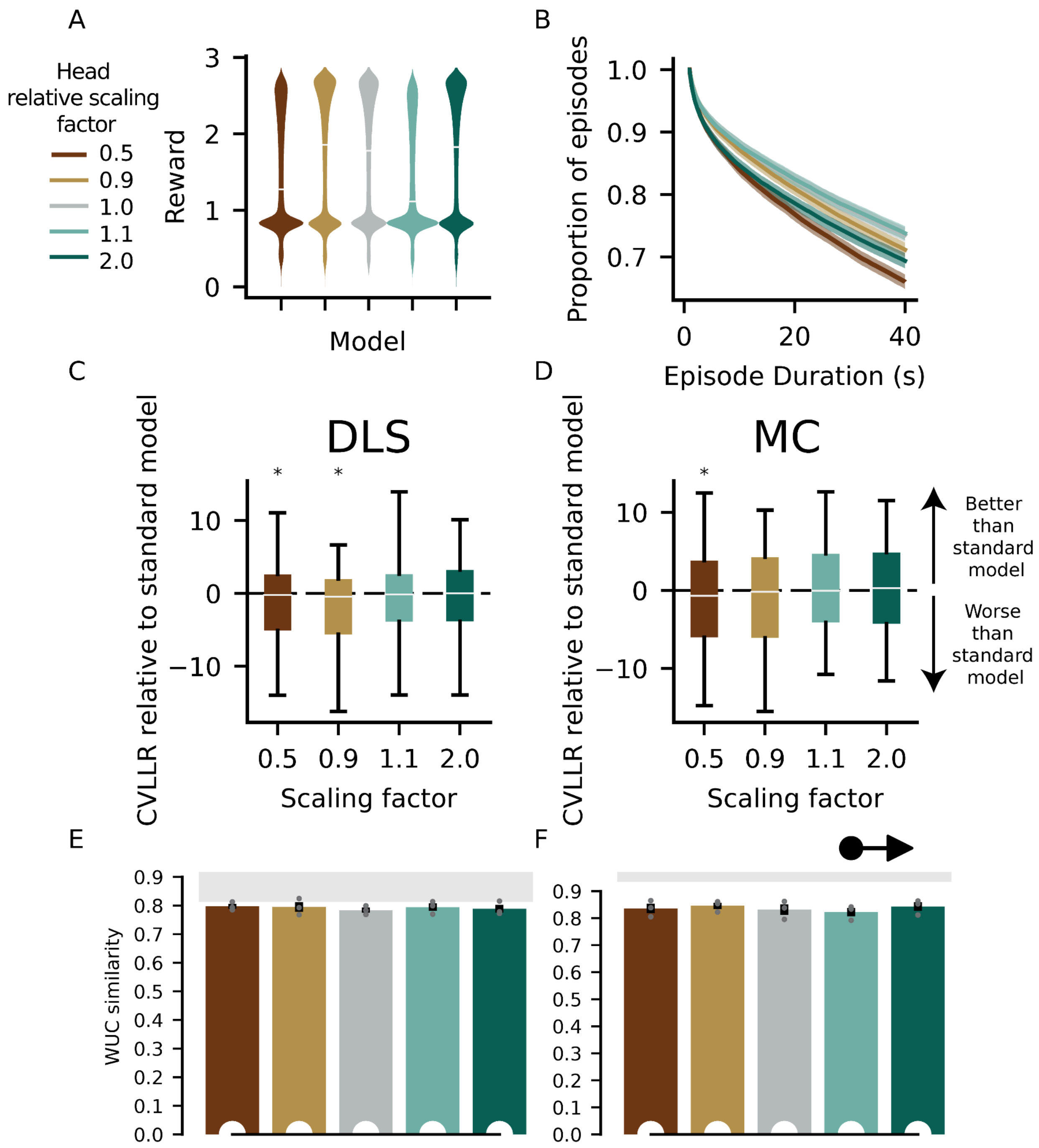

A
Head relative scaling factor
0.5
0.9
1.0
1.1
2.0
Reward
Model
B
Proportion of episodes
Episode Duration (s)
C
DLS
CVLLR relative to standard model
Scaling factor
D
MC
Better than standard model
Worse than standard model
E
WUC similarity
F

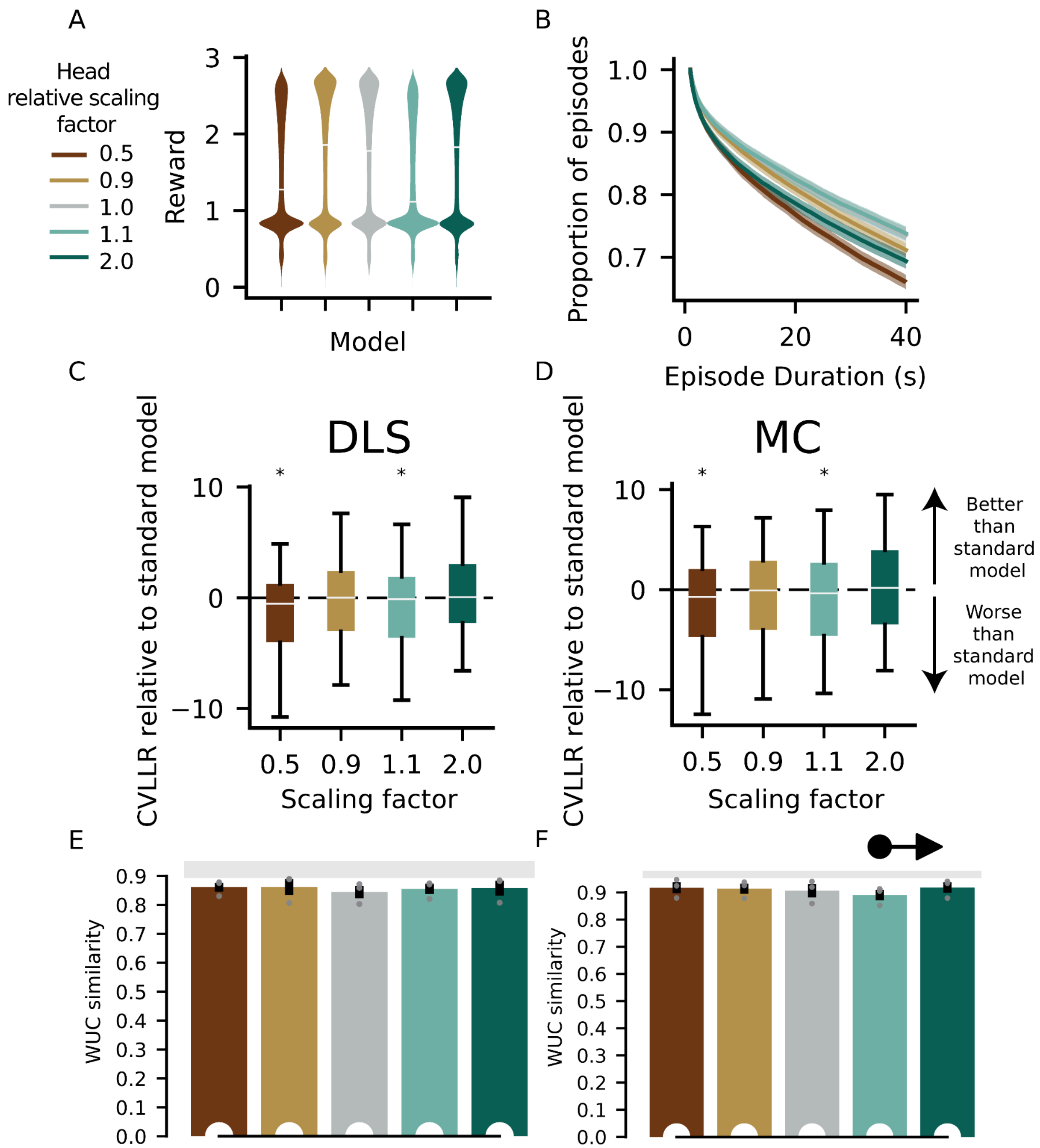
A
Head relative scaling factor
0.5
0.9
1.0
1.1
2.0
Reward
Model
B
Proportion of episodes
Episode Duration (s)
C
DLS
CVLLR relative to standard model
Scaling factor
D
MC
Better than standard model
Worse than standard model
E
WUC similarity
F

Extended Data Figure 9

Panels D-I have been updated to include the corrected data. For panels F and G, there were differences in the significance of the multiple comparisons tests between the original and updated figures. The results remain consistent with our conclusions.

The original figure appears on top, while the corrected figure appears on the bottom.

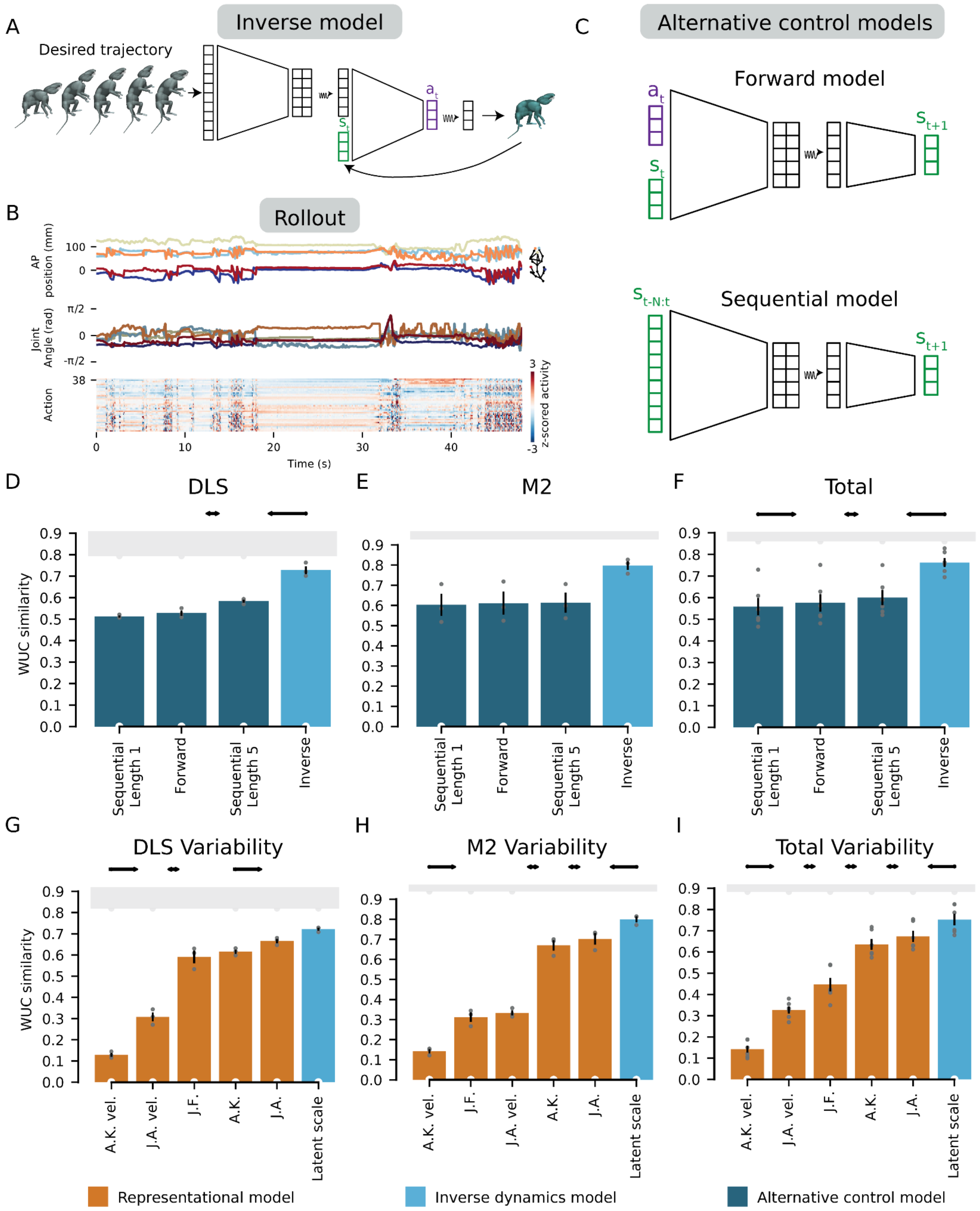
A
Inverse model
Desired trajectory
C
Alternative control models
Forward model
Sequential model
B
Rollout
AP position (mm)
Joint Angle (rad)
Action
z-scored activity
Time (s)
D
DLS
E
M2
F
Total
WUC similarity
Sequential Length 1
Forward
Sequential Length 5
Inverse
G
DLS Variability
H
M2 Variability
I
Total Variability
A.K. vel.
J.A. vel.
J.F.
A.K.
J.A.
Latent scale
Representational model
Inverse dynamics model
Alternative control model

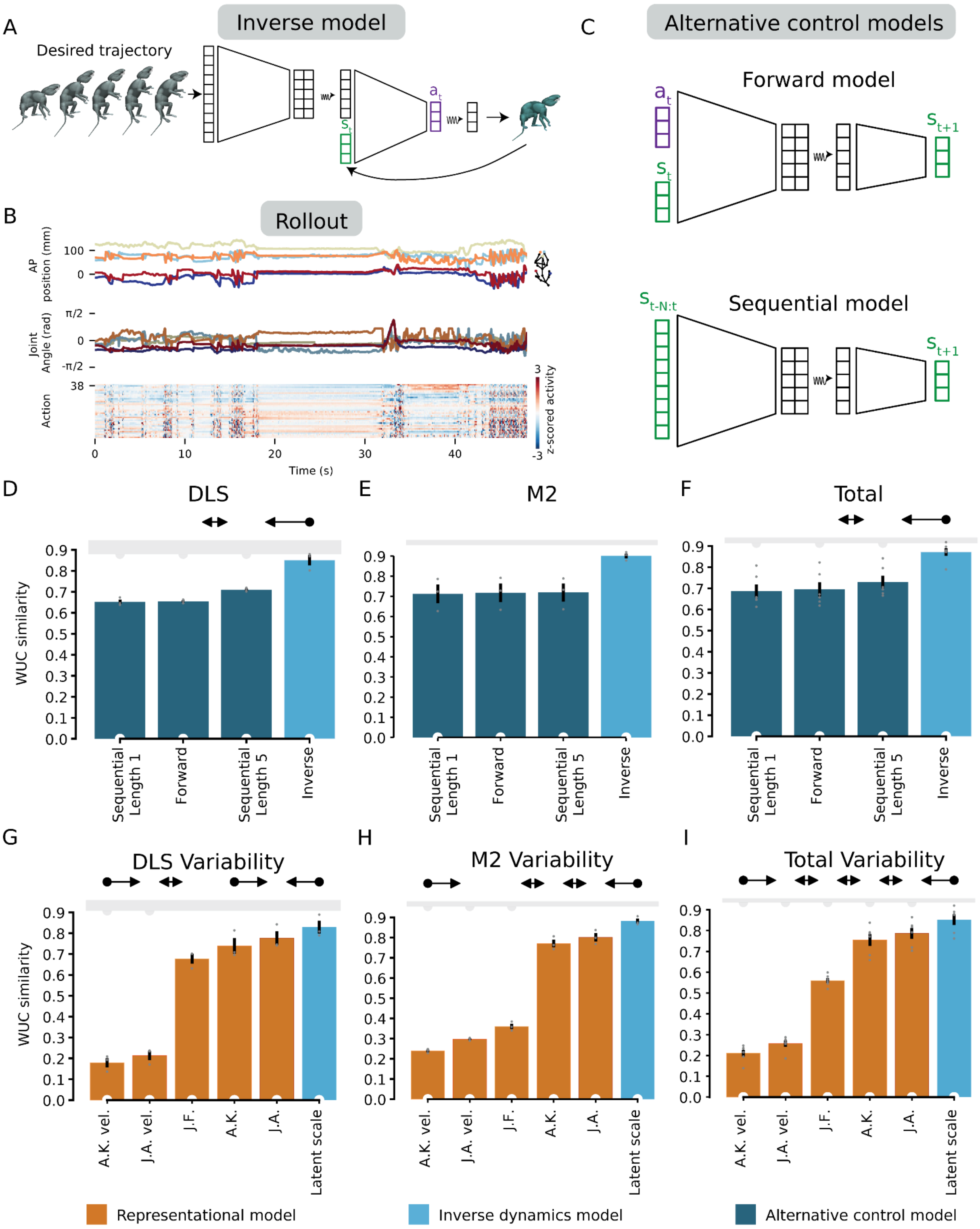

A
Inverse model
Desired trajectory
C
Alternative control models
Forward model
Sequential model
B
Rollout
AP position (mm)
Joint Angle (rad)
Action
Time (s)
z-scored activity
D
DLS
WUC similarity
E
M2
F
Total
Sequential Length 1
Forward
Sequential Length 5
Inverse
G
DLS Variability
H
M2 Variability
I
Total Variability
A.K. vel.
J.A. vel.
J.F.
A.K.
J.A.
Latent scale
Representational model
Inverse dynamics model
Alternative control model

Extended Data Figure 10

All panels have been updated to include the corrected data. The distributions of CVLLRs for neurons in the striatum and motor cortex remain similar after the update, including for alternative control models.

The original figure appears on top, while the corrected figure appears on the bottom.

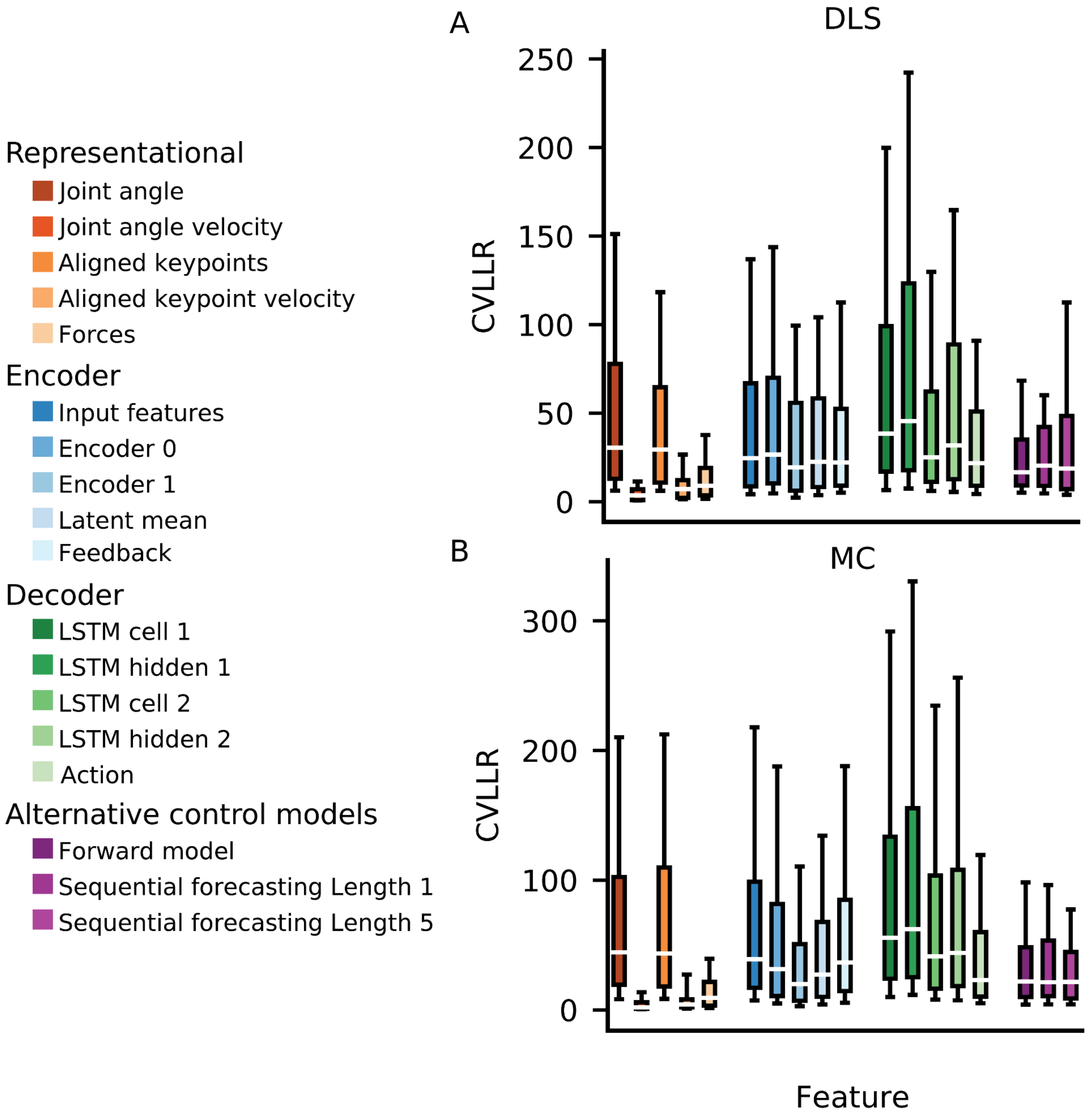

Representational
Joint angle
Joint angle velocity
Aligned keypoints
Aligned keypoint velocity
Forces
Encoder
Input features
Encoder 0
Encoder 1
Latent mean
Feedback
Decoder
LSTM cell 1
LSTM hidden 1
LSTM cell 2
LSTM hidden 2
Action
Alternative control models
Forward model
Sequential forecasting Length 1
Sequential forecasting Length 5
A
DLS
250
200
150
100
50
0
CVLLR
B
MC
300
200
100
0
CVLLR
Feature

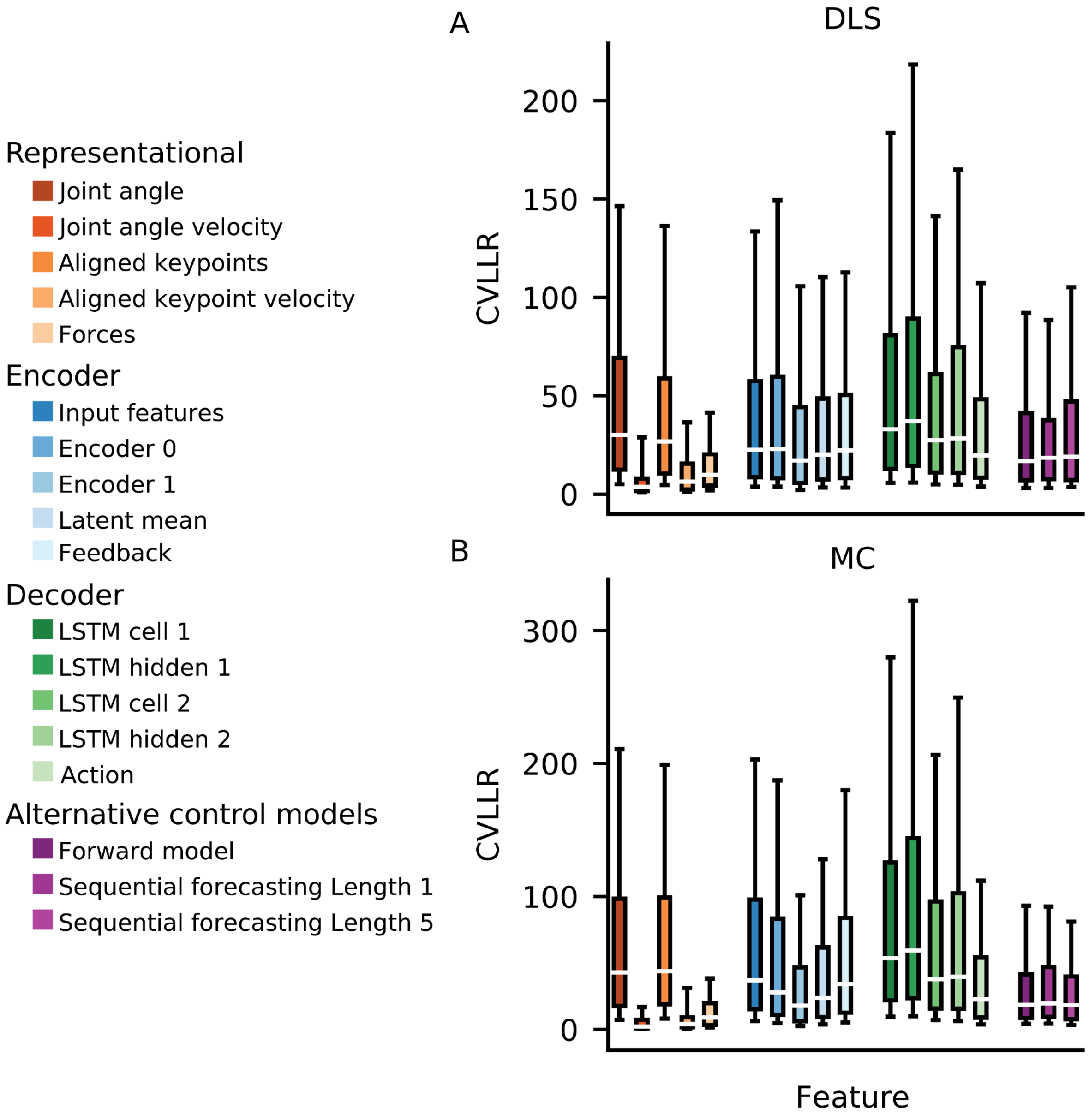

Representational
Joint angle
Joint angle velocity
Aligned keypoints
Aligned keypoint velocity
Forces
Encoder
Input features
Encoder 0
Encoder 1
Latent mean
Feedback
Decoder
LSTM cell 1
LSTM hidden 1
LSTM cell 2
LSTM hidden 2
Action
Alternative control models
Forward model
Sequential forecasting Length 1
Sequential forecasting Length 5
A
DLS
CVLLR
0
50
100
150
200
B
MC
CVLLR
0
100
200
300
Feature
